# Empiric anti-Giardia therapy in non-diarrheal protein-losing enteropathy: A focus on children with monogenic humoral immunodeficiency

**DOI:** 10.5339/qmj.2023.sqac.24

**Published:** 2023-11-26

**Authors:** Tariq Al Farsi, >Khwater Ahmed, Nashet Al Sukaiti

**Affiliations:** ^1^Pediatric allergy and clinical immunology, The Royal Hospital. Muscat-Oman Email: alfarsitq@gmail.com

## Abstract

**Background and Aim:** Chronic giardia infection can lead to non-erosive gastrointestinal disorders, including protein-losing enteropathy (PLE). This report describes non-diarrheal PLE in chronic giardiasis in children with defective humoral immunity.

Methods: The retrospective report is related to 2 children known to have a monogenic inborn error of immunity. The first patient is a 13-year-old boy with X-linked agammaglobulinemia (Patient-1), and the second is a 5-year-old boy with NF-kB inducing kinase (NIK) deficiency infection. Frequency, growth status, and serum immunoglobulin-G (IgG) trough and albumin levels were monitored (Patient-2).

**Results:** Patient-1 had more frequency of pneumonia but reported no symptoms of gastrointestinal disease, including alteration of bowel habits, change in stool consistency, nausea, vomiting, fatigue, bloating, and abdominal pain. No clinical oedema on examination. His weight remains static at 19-20 kg for about 1.5 years. Simultaneously, hypoproteinaemia was noted (Figure-1).

A trial of increasing IVIG (0.7 – 1 g/kg) and the use of subcutaneous immunoglobulin (0.2 g/kg) did not reverse the biochemical and clinical situation. Hypoproteinaemia workup revealed normal liver and kidney functions, normal cardiac function, and no proteinuria. Interestingly, his upper endoscopy showed mild duodenitis and the presence of giardia lamblia at the luminal surface (Figure-2).

Following this, a 2-week course of oral metronidazole (7.5 mg/kg/dose BID) resulted in a restoration of therapeutic serum IgG trough and albumin levels. Additionally, the child’s nutritional status improved, and the frequency of respiratory infections dropped. In Patient-2, progressive hypoproteinaemia was noted over nine months. Similarly, no gastrointestinal complaints; however, the family reported foul-smelling semisolid stools with regular consistency. His IgG trough level was 2 g/l, and his albumin level was 20 g/l. Despite maximizing IVIG, he developed two episodes of pneumonia and once otitis media. An empiric oral metronidazole course resulted in the amelioration of symptoms and restoration of proteinemia. Meanwhile, faeces microscopy confirmed the presence of Giardia lamblia.

**Conclusions:** Progressive hypoproteinaemia in children with humoral immunodeficiency is a clinical concern. Routine stool microscopy can identify giardia infection despite the ambiguity of gastrointestinal symptoms in such patients. However, a trial of empiric metronidazole therapy should be considered.

## Figures and Tables

**Figure 1.**
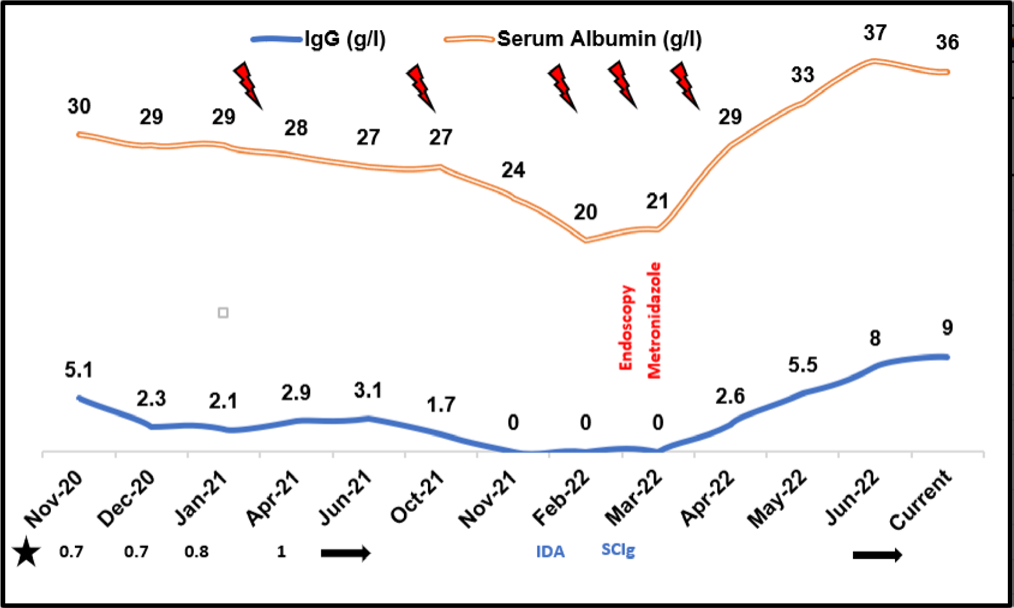
The line chart describes serum IgG trough and albumin levels over the observation period. IDA: iron deficiency anaemia, SCIg: subcutaneous immunoglobulin. Symbol: bacterial pneumonia, ★ 3-weekly IVIG dose (g/kg).

**Figure 2.**
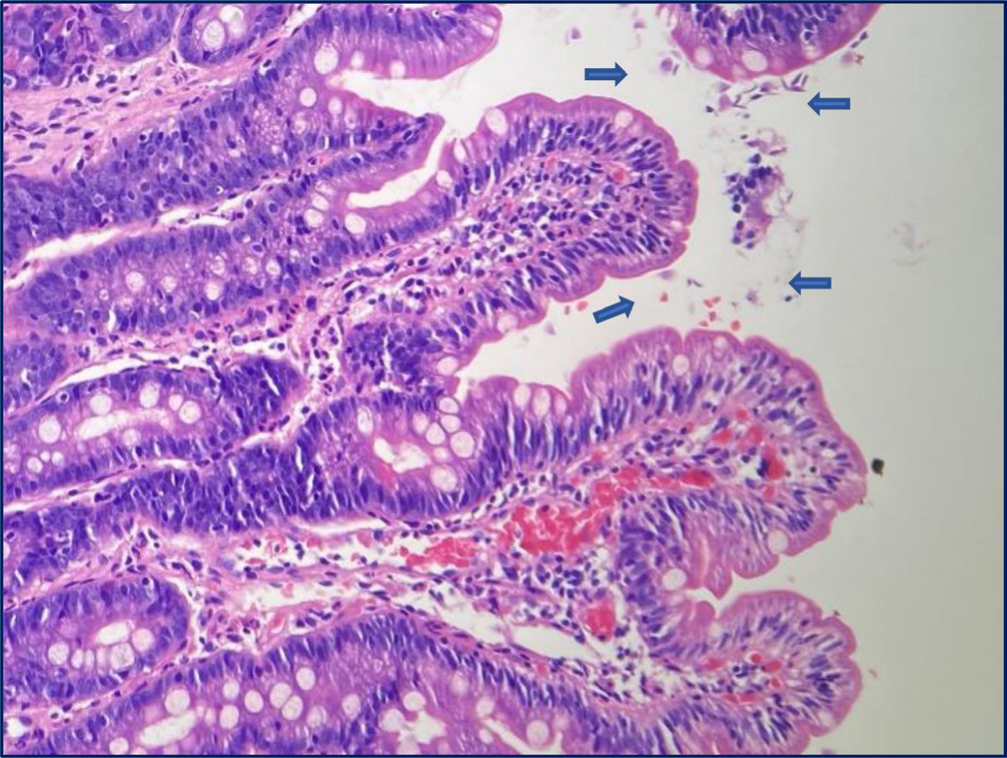
High power, H & E stain of duodenal mucosa showing absent plasma cells, mild duodenitis, and multiple cysts and trophozoites of giardia lamblia (arrows).
